# Supplementary material for: Effects of Probiotic Supplementation on Body Weight, Growth Performance, Immune Function, Intestinal Microbiota and Metabolites in Fallow Deer
Source: Biology (Basel). 2024 Aug 9;13(8):603. doi: 10.3390/biology13080603 (PMC11351348; doi:10.3390/biology13080603)
Supplement: Supplementary file 1 [file biology-13-00603-s001.zip › biology-3095811-supplementary.pdf]

Table S1. Mixed factorial ANOVA and *F* statistics for body weight.

Construct	Group	Mean±SEM				Test Effect	p-Valu[illegible]
		0d	35d	65d	170d		
Body weight (g)	Control	11.62±1.00	12.15±0.71	17.39±0.95	25.84±1.23	Time<br>Group	< 0.01[illegible]<br>0.022
	Treatment	12.17±0.55	16.58±0.63***	21.40±0.54***	28.46±0.89*	Time*Group	< 0.01[illegible]

Values are expressed as mean ± SEM, with different superscripts in the rows indicating a significant difference compared to the control group, * [illegible] 0.001.

Table S2. Mixed factorial ANOVA and *F* statistics for other assays.

Construct	Group	Mean±SEM			Test Effect	*p*-Value	η^2
		35d	65d	170d			
Gain weight (g)	Control	0.53±0.74	5.77±0.14	14.23±0.68	Time	<0.001	0.973
					Group	<0.001	0.770
	Treatment	4.42±0.39***	9.23±0.50***	16.29±0.62*	Time*Group	0.186	0.164
Daily weight Gain (g)	Control	0.02±0.02	0.09±0.02	0.22±0.01	Time	<0.001	0.924
					Group	<0.001	0.781
	Treatment	0.13±0.01***	0.14±0.01***	0.25±0.01*	Time*Group	0.012	0.442
Serum IgA (g/L)	Control	1.24±0.06	1.21±0.02	1.22±0.04	Time	0.262	0.156
					Group	0.004	0.676
	Treatment	1.24±0.18	1.55±0.10*	1.50±0.02*	Time*Group	0.176	0.207
Serum IgM (g/L)	Control	0.75±0.03	0.74±0.04	0.88±0.08	Time	0.281	0.119
					Group	0.005	0.568
	Treatment	0.97±0.03***	0.99±0.10*	1.03±0.08	Time*Group	0.769	0.024
Serum IgG (g/L)	Control	7.64±0.53	8.23±0.36	8.16±0.17	Time	0.555	0.052
					Group	0.040	0.391
	Treatment	9.44±0.67	8.99±0.34	8.35±0.10	Time*Group	0.152	0.203

Values are expressed as mean ± SEM, with different superscripts in the rows indicating a significant difference compared to the control group, * for p < 0.05, ** for p < 0.01 and *** for p < 0.001.
